# The recognition of time-compressed speech as a function of age in listeners with cochlear implants or normal hearing

**DOI:** 10.3389/fnagi.2022.887581

**Published:** 2022-09-29

**Authors:** Anna R. Tinnemore, Lauren Montero, Sandra Gordon-Salant, Matthew J. Goupell

**Affiliations:** ^1^Neuroscience and Cognitive Science Program, University of Maryland, College Park, College Park, MD, United States; ^2^Department of Hearing and Speech Sciences, University of Maryland, College Park, College Park, MD, United States

**Keywords:** cochlear implant, time compression, aging, speech perception, temporal processing, fast speech, behavior, hearing loss

## Abstract

Speech recognition is diminished when a listener has an auditory temporal processing deficit. Such deficits occur in listeners over 65 years old with normal hearing (NH) and with age-related hearing loss, but their source is still unclear. These deficits may be especially apparent when speech occurs at a rapid rate and when a listener is mostly reliant on temporal information to recognize speech, such as when listening with a cochlear implant (CI) or to vocoded speech (a CI simulation). Assessment of the auditory temporal processing abilities of adults with CIs across a wide range of ages should better reveal central or cognitive sources of age-related deficits with rapid speech because CI stimulation bypasses much of the cochlear encoding that is affected by age-related peripheral hearing loss. This study used time-compressed speech at four different degrees of time compression (0, 20, 40, and 60%) to challenge the auditory temporal processing abilities of younger, middle-aged, and older listeners with CIs or with NH. Listeners with NH were presented vocoded speech at four degrees of spectral resolution (unprocessed, 16, 8, and 4 channels). Results showed an interaction between age and degree of time compression. The reduction in speech recognition associated with faster rates of speech was greater for older adults than younger adults. The performance of the middle-aged listeners was more similar to that of the older listeners than to that of the younger listeners, especially at higher degrees of time compression. A measure of cognitive processing speed did not predict the effects of time compression. These results suggest that central auditory changes related to the aging process are at least partially responsible for the auditory temporal processing deficits seen in older listeners, rather than solely peripheral age-related changes.

## Introduction

Cochlear implants (CIs) are auditory prostheses that only convey partial speech information to listeners via a series of electrical pulses across a limited number of electrode contacts. Although this highly distorted rendition of sound is sufficient for most listeners to recognize speech with varying degrees of success in quiet environments (Gifford et al., 2008), real-world listening conditions are frequently less than ideal. While CIs faithfully convey some aspects of acoustic speech, specifically temporal envelope cues, CI processing distorts or eliminates other aspects. Other forms of distortion, such as rapid or time-compressed speech, can result in further deterioration in speech recognition for adults with CIs (Fu et al., 2001; Ji et al., 2013). Recognition of rapid or time-compressed speech is also difficult for older adult listeners with normal hearing (NH) (e.g., Tun, 1998; Gordon-Salant and Fitzgibbons, 2001; Golomb et al., 2007). The age-related difficulty in recognizing rapid or time-compressed speech is at least partially related to deficits in basic auditory temporal processing abilities, such as duration discrimination and gap detection (e.g., Gordon-Salant and Fitzgibbons, 1993; Fitzgibbons and Gordon-Salant, 1996; Gordon-Salant et al., 2006). Thus, there are many types of distortions that can affect speech understanding. Three types of distortion are considered in the present study: distortion imposed by CI sound processing, distortion of the input (rapid speech), and distortion caused by aging neural and cognitive systems responsible for processing temporal speech cues. These distortions have known individual effects on speech recognition; however, how these factors affect older listeners with CIs and interact with each other are as yet unknown.

The first type of distortion, the sound processing of the CI, is inherent to the limitations of the technology. Acoustic sound is processed and transduced by a CI into electrical pulses that are transmitted to the listener’s auditory nerve. In this electrical signal, temporal envelope information is largely maintained (Wouters et al., 2015), but temporal fine structure and spectral resolution are greatly reduced (Friesen et al., 2001). With time after activation, a listener with a CI often improves in speech understanding performance (Blamey et al., 2013). Some of this improvement is thought to result from the listener’s adaptation to speech that has reduced spectral detail and no temporal fine structure. A simulation of CI-processed speech can be created by eliminating the acoustic fine structure and conveying the temporal envelope using a limited number of channels, as in Friesen et al. (2001). These researchers compared the performance of listeners with CIs and various numbers of electrodes activated to that of listeners with NH and various numbers of channels in simulations of CI-processed speech (i.e., vocoded speech). They found that while CIs typically have 12–24 electrodes, the effective spectral resolution lies between 8 and 10 channels because of the spread of excitation in the cochlea (Friesen et al., 2001). Using vocoded speech allows researchers to present listeners with NH a signal that has been processed in a similar manner to that available to listeners with CIs.

The second type of distortion, rapid or time-compressed speech, disrupts the speech recognition of older listeners with NH more than that of younger listeners with NH (e.g., Konkle et al., 1977; Gordon-Salant and Fitzgibbons, 1993; Tun, 1998; Wingfield et al., 2003). These studies used varying stimuli, from monosyllabic words to complete sentences. In all of them, performance decreased for all listener age groups as the rate of time compression increased. The oldest groups consistently demonstrated larger decreases in performance compared to the younger age groups. As noted above, adults with CIs, both younger and older, also experience difficulty understanding time-compressed speech. Time-compressed speech is not a perfect analog to naturally produced rapid speech. In fact, recognition of time-compressed speech is often better than recognition of naturally produced rapid speech of the same rate (e.g., Gordon-Salant et al., 2014). However, time-compressed speech is a useful tool for examining the effect of temporal rate changes on listeners who rely on the temporal envelope to understand speech. Further, the potential age-related changes in the ability to recognize time-compressed speech in listeners with CIs is not yet known.

The third type of distortion, age-related changes to neural and cognitive mechanisms responsible for processing temporal speech cues, can affect speech recognition when the speech signal is distorted or background noise is present (e.g., Füllgrabe et al., 2015; Babkoff and Fostick, 2017). Age-related declines in speech recognition have been attributed to declines in peripheral sensitivity, central processing, and/or cognitive abilities (Working Group on Speech Understanding and Aging, 1988). Peripheral hearing loss is prevalent among older adults (Cruickshanks et al., 1998; Lin et al., 2011) and corresponds with declines in speech understanding (e.g., Humes and Dubno, 2010). Age-related reductions in central processing abilities, such as auditory temporal processing, can be linked to age-related changes in the brain, such as reductions in myelination on the auditory nerve and alterations to response properties of neurons (e.g., Gates et al., 2008; Canlon et al., 2010). These central processing changes have been shown to correspond with poorer understanding of speech that is distorted or presented in background noise (e.g., Humes et al., 2012; Presacco et al., 2016). Additionally, cognitive abilities that commonly change with age include reductions in processing speed (Salthouse, 2000), working memory (e.g., Zekveld et al., 2013), and inhibition (e.g., Hasher et al., 1991). Reduced cognitive abilities in these domains have also been linked to poorer speech understanding in background noise (e.g., Rönnberg et al., 2010; Rudner et al., 2011). When relying solely on temporal cues for speech communication, such as when using a CI to hear, it is possible that central and cognitive abilities may be crucial to support speech understanding.

Multiple cognitive abilities, such as working memory and processing speed, have been shown to correlate with the ability of older adults to understand rapid speech (e.g., Wingfield et al., 2003; Vaughan et al., 2006; Dias et al., 2019). Studying the speech perception abilities of older adults with CIs may allow researchers to determine the relative contributions of these cognitive factors to the ability to recognize rapid speech. Several studies have documented improved speech perception with the use of CIs in older adults (e.g., Dillon et al., 2013; Forli et al., 2019; Canfarotta et al., 2020; Murr et al., 2021). Despite the clear benefits of CIs for understanding normal-rate speech in quiet, less is known about the performance of older adults using a CI in more demanding listening situations. Thus, evaluating speech recognition of adults of varying ages who use CIs to recognize challenging speech materials will provide a more realistic picture of the daily communication challenges faced by listeners with CIs, as well as insight into the underlying peripheral, central, and cognitive mechanisms that contribute to these difficulties.

A common issue in previous studies investigating the effect of age on auditory tasks is the confounding factor of peripheral age-related hearing loss. This hearing loss may impact older listeners’ performance despite all the listeners having “clinically normal hearing” or “normal hearing for their age” through a certain subset of audiometric frequencies (e.g., Gelfand et al., 1986; Takahashi and Bacon, 1992; Shader et al., 2020b; Lentz et al., 2022). For example, Shader et al. (2020b) reported that the younger listeners with normal hearing had significantly lower (better) thresholds than the older listeners with normal hearing; these hearing acuity differences were the main source of age-related differences in recognition of noise-vocoded sentences. This confound should be reduced by testing listeners with CIs, because the CI bypasses many of the outer, middle, and inner ear sources of age-related hearing loss. In theory, older listeners with CIs are receiving the same peripheral signals as younger listeners with CIs, the main difference being age-related loss of spiral ganglia that could cause differences in neural survival and in the electrode-to-neuron interface (Makary et al., 2011).

If the documented age-related deficit for recognizing time-compressed speech is primarily a result of cochlear hearing loss, then a comparison between younger and older listeners with CIs would not show an age-related deficit, because both groups would be using a device that bypasses cochlear encoding. Alternatively, if the source of the age-related deficit for recognizing time-compressed speech is primarily a result of central auditory or cognitive processing changes, then a comparison between younger and older listeners with CIs would show an age-related deficit similar to that observed for listeners with NH presented with a CI simulation. In other words, older listeners would show the same age-related deficits compared to younger listeners regardless of whether they listen with a CI or to a CI simulation.

The current study was conducted with two listener groups: those who use CIs and those with NH who were presented a CI simulation (vocoded speech). The listeners with NH were included as a control group for comparison to the listeners with CIs, using the same speech materials and time compression methods. Additionally, listeners in both groups were recruited in three age categories (younger, middle-aged, and older) in order to provide insight into the age at which time-compressed speech recognition deficits become apparent and to facilitate comparison of the main patterns of performance between the types of listeners (CI, vocoded speech) across the adult age span. The first hypothesis was that there would be age-related decreases in recognizing time-compressed speech by both listeners with CIs and listeners with NH presented with vocoded speech. This age-related decrease in speech recognition was hypothesized to be larger with greater degrees of time compression (i.e., there would be an age group by degree of time compression interaction). The second hypothesis was that faster cognitive processing speed [as measured by the Wechsler Adult Intelligence Scale (WAIS) (Wechsler, 1955)] would be predictive of better performance in recognizing time-compressed speech. Such a result would support the notion that cognitive decline is a significant source of the age-related decrease in recognizing time-compressed speech.

## Materials and methods

### Listeners

**FIGURE 1:**
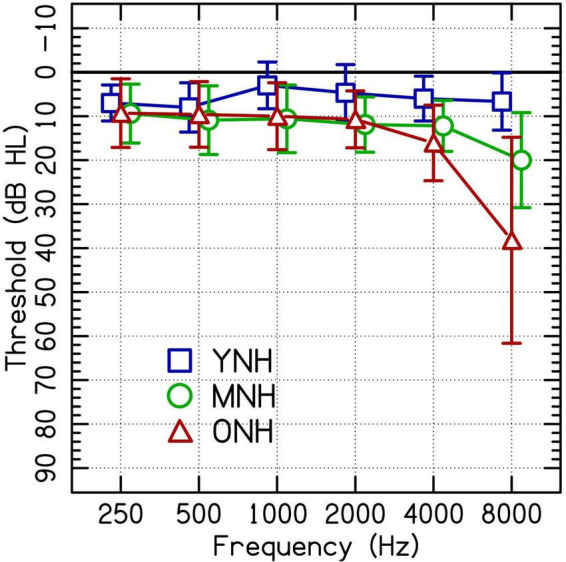
Group average audiometric thresholds of the test ears of participating listeners with clinically normal hearing at audiometric frequencies between 250 and 4,000 Hz separated into younger (YNH), middle-aged (MNH), and older (ONH) age groups. Error bars are ± 1 SD.

A total of 46 listeners with NH were assigned to one of three groups by age: younger, middle-aged, and older. The younger listeners with NH (YNH; *n* = 15) were 19–23 years old (*M* = 20.67, SD = 1.23). The middle-aged listeners with NH (MNH; *n* = 16) were 52–64 years old (*M* = 59.75, SD = 2.74). The older listeners with NH (ONH; *n* = 15) were 65–78 years old (*M* = 69.79, SD = 4.17). All listeners with NH had thresholds ≤25 dB HL (American National Standards Institute/Acoustical Society of America [ANSI/ASA], 2018) at audiometric test frequencies from 250 to 4,000 Hz in at least the better-hearing ear. See Figure 1 for audiometric data.

A total of 58 listeners with CIs were also assigned to one of three age groups: younger, middle-aged, and older. The younger listeners with CIs (YCI: *n* = 16) were 20–48 years old (*M* = 33.1, SD = 9.35). The middle-aged listeners with CIs (MCI; *n* = 21) were 50–63 years old (*M* = 55.1, SD = 4.12). The older listeners with CIs (OCI; *n* = 21) were 65–82 years old (*M* = 71.5, SD = 4.85). See Table 1 for demographic information such as the length of time between when a listener lost usable hearing and implantation (duration of deafness), the duration of implantation, CI processor, and whether the listener mostly learned spoken language before or after implantation.

**TABLE 1:** Demographic information for listeners with CIs.

Subject code	Age (years)	Ear Tested	Duration of deafness (in ear tested) (years)	Duration of implantation (years)	Processor	Onset of hearing loss (Pre-/post-lingual)
**Younger listeners with CIs (YCI)**						
YCI001	23	R	1	5	Freedom	Post-lingual
YCI002	21	R	3	18	Harmony	Pre-lingual
YCI003	20	R	2	18	N6 (CP910)	Pre-lingual
YCI004	24	L	<1	2	N6 (CP910)	Post-lingual
YCI005	36	L	3	16	N5 (CP810)	Post-lingual
YCI006	41	L	40	1	Naida	Post-lingual
YCI007	41	R	1	1	Naida Q70	Post-lingual
YCI008	42	R	5	14	N5 (CP810)	Post-lingual
YCI009	48	R	<1	0.5	Naida Q70	Post-lingual
YCI010	30	R	<1	28	N6 (CP910)	Post-lingual
YCI011	35	R	<1	1	Sonnet, Rondo	Post-lingual
YCI012	43	L	37	5	N6 (CP910)	Pre-lingual
YCI013	21	R	2	19	Opus 2	Pre-lingual
YCI014	27	R	8	9	N6 (CP910)	Post-lingual
YCI015	45	R	24	21	N6 (CP920)	Pre-lingual
YCI016	32	R	<1	30	N6 (CP910)	Pre-lingual
**Middle-aged listeners with CIs (MCI)**						
MCI001	56	L	11	5	N6 (CP910)	Post-lingual
MCI002	54	L	2	52	N5 (CP810)	Post-lingual
MCI003	53	L	<1	4	N6 (CP910)	Post-lingual
MCI004	57	R	<1	9	N5 (CP810)	Post-lingual
MCI005	54	L	31	2	N5 (CP810)	Pre-lingual
MCI006	57	L	5	5	N6 (CP920)	Post-lingual
MCI007	61	R	2	4	Kanso (CP950)	Post-lingual
MCI008	55	L	1	2	Rondo, Opus 2	Post-lingual
MCI009	52	R	33	2	Opus 2	Post-lingual
MCI010	56	L	25	6	N6 (CP910)	Post-lingual
MCI011	50	L	10	8	Harmony	Post-lingual
MCI012	50	R	2	11	N5 (CP810)	Post-lingual
MCI013	62	R	13	4	N6 (CP910)	Post-lingual
MCI014	62	L	11	3	N5 (CP810)	Post-lingual
MCI015	63	R	<1	8	Naida Q70	Post-lingual
MCI016	51	L	<1	7	N6 (CP910)	Post-lingual
MCI017	55	R	<1	14	N6 (CP920)	Post-lingual
MCI018	55	L	5	12	Harmony	Post-lingual
MCI019	54	L	6	8	N6 (CP910)	Post-lingual
MCI020	50	R	20	7	N5 (CP810)	Post-lingual
MCI021	50	R	8	2	N5 (CP810)	Post-lingual
**Older listeners with CIs (OCI)**						
OCI001	71	L	36	20	N5 (CP810)	Post-lingual
OCI002	76	L	7	7	N6 (CP910/920)	Post-lingual
OCI003	65	L	2	10	N6 (CP920)	Post-lingual
OCI004	71	R	4	12	N5 (CP810)	Post-lingual
OCI005	68	R	<1	10	N6 (CP920)	Post-lingual
OCI006	70	R	4	8	N6 (CP910)	Post-lingual
OCI007	70	R	<1	5	N5 (CP810)	Post-lingual
OCI008	82	R	2	3	N5 (CP810)	Post-lingual
OCI009	77	L	<1	1	N6 (CP920)	Post-lingual
OCI010	79	R	<1	5	Freedom	Post-lingual
OCI011	65	L	12	8	N6 (CP920)	Post-lingual
OCI012	65	R	5	<1	Naida	Post-lingual
OCI013	70	R	5	4	N5 (CP810)	Post-lingual
OCI014	69	R	12	15	Naida Q70	Post-lingual
OCI015	77	R	1	6	N7 (CP1000)	Post-lingual
OCI016	65	R	8	3	N6 (CP910)	Post-lingual
OCI017	73	R	4	20	Naida Q90	Post-lingual
OCI018	72	R	<1	12	N7 (CP1000)	Post-lingual
OCI019	69	R	<1	7	N5 (CP810)	Post-lingual
OCI020	72	L	<1	4	N6 (CP910)	Post-lingual
OCI021	76	R	29	7	N5 (CP810)	Post-lingual

The age groups were not quite evenly matched. The ages of the younger listeners with NH were significantly lower than the ages of the younger listeners with CIs [*t*(15.5) = −5.13, *p* < 0.001; two-tailed independent samples *t*-test]. The ages of the middle-aged listeners with NH were significantly higher than the ages of the middle-aged listeners with CIs [*t*(34.5) = 4.11, *p* < 0.001; two-tailed independent samples *t*-test]. The ages of the older listeners with NH were not significantly different than the ages of the older listeners with CIs [*t*(33.2) = −1.16, *p* > 0.05; two-tailed independent samples *t*-test]. The well-matched older groups were vital for drawing valid conclusions about the presence or absence of any age-related deficits across listener groups.

### Stimuli

The stimuli were Institute of Electrical and Electronic Engineers (IEEE) sentences (Rothauser, 1969) spoken by a male, native speaker of American English. Each sentence has five keywords. Sentences were time-compressed using the PSOLA algorithm in Praat version 5.3.56 (Boersma and Weenink, 2013), which removes minute portions of the waveform at set intervals before condensing the remaining waveform together. This method maintains the speech envelope and many of the pitch characteristics of the original speech. Sentences were compressed by 0% (i.e., no time compression), 20, 40, and 60%. A sentence compressed by 40% has a duration equal to 60% of the original length. At 0% time compression, the talker spoke at an average rate of approximately 3.7 syllables per second. The rate increased to approximately 4.6 syllables per second in the 20% time-compressed sentences, approximately 6.4 syllables per second in the 40% time-compressed sentences, and approximately 10 syllables per second in the 60% time-compressed sentences. These time-compressed sentences were used as the stimuli for the listeners with CIs.

For listeners with normal hearing, the sentences at all four degrees of time compression were also vocoded into 16, 8, and 4 channels using noise vocoding (Shannon et al., 1995). For an *n*-channel vocoder, pre-emphasis was added to the auditory speech signal using a 1st-order forward Butterworth high-pass filter at 1,200 Hz. The pre-emphasized auditory speech signal was then bandpass filtered using 3rd-order forward-backward Butterworth filters into *n* logarithmically spaced bands (36 dB/octave) between 200 and 8,000 Hz. The temporal speech envelope from each band was extracted with a Hilbert envelope cutoff of 160 Hz and then used to modulate *n* noise carriers that were bandpass filtered to match the width of the *n* logarithmically spaced bands. The 16, 8, or 4 modulated noise carriers were then combined to create the final vocoded output.

### Procedure

All procedures were conducted with the informed consent of the listeners and were approved by the Institutional Review Board of the University of Maryland. Listeners were compensated for their time and participation.

#### Preliminary measures and cognitive assessments

Air conduction thresholds were measured for each NH listener in a sound-treated booth using a Maico MA41 audiometer and TDH-39 headphones. All listeners completed the Montreal Cognitive Assessment (MoCA) (Nasreddine et al., 2005) as a screener for study participation. Listeners with NH had to score 26 or higher (out of 30 possible) in order to proceed, while listeners with CIs had to score 22 or higher because of the confounds of giving a screening in a modality in which the person struggles (Dupuis et al., 2015). Each listener also completed a standardized subtest from the Wechsler Adult Intelligence Scale (WAIS III) (Wechsler, 1955) to measure speed of processing: the Symbol Search test. In the Symbol Search test, two sets of symbols were shown to the listener. The first set consisted of two symbols and the second set consisted of five symbols. The listener had to mark “Yes” if either of the two symbols in the first set were present in the second set and “No” if neither symbol occurred in the second set. They had 2 minutes to complete as many sets as they could. Listeners were instructed to perform the task as quickly and accurately as possible. They were scored on the number of items correctly completed in the allotted time.

#### Training on vocoded stimuli for listeners with normal hearing

Listeners with NH completed a training phase to familiarize them with vocoded speech. Stimuli used during training were low-context sentences created from a closed set of monosyllabic words. Each sentence contained a name, a verb, a number, an adjective, and a noun. For example, “Pat saw two red bags” or “Jill took five small hats” (Kidd et al., 2008). Sentences were recorded by a male talker at his normal rate of speech and were vocoded following the same procedure as the experimental sentences into 16, 8, or 4 channels. During training, listeners heard three blocks of 15 vocoded sentences drawn randomly from the 16-, 8-, and 4-channel vocoded sentences. Listeners were seated in front of a computer in a double-walled sound-attenuated booth (Industrial Acoustics Inc., Bronx, NY, USA). The sentences were presented through a soundcard (UA-25 EX, Edirol/Roland Corp., Los Angeles, CA, USA) and amplifier (D-75A, Crown Audio, Elkhart, IN, USA) monaurally over circumaural headphones (Sennheiser HD 650, Hanover, Germany). The ear with better hearing was chosen for this experiment, or the right ear if thresholds were the same in the two ears. MATLAB software (MathWorks, Natick, MA, USA) was used to present a five-by-eight grid of words on the computer screen. Each sentence contained one of the eight words from each column. The listener selected the words that they heard in each sentence, guessing if they were unsure. Visual feedback was provided after each trial. After completing the 45 sentences of practice, listeners started the experimental protocol.

#### Experimental protocol

All listeners were seated comfortably in a sound-attenuating booth (Industrial Acoustics Inc., Bronx, NY, USA). Listeners with normal hearing used circumaural headphones (Sennheiser HD 650, Hanover, Germany) to listen to stimuli presented at 75 dB(A) through a soundcard (UA-25 EX, Edirol/Roland Corp., Los Angeles, CA, USA) and amplifier (D-75A, Crown Audio, Elkhart, IN, USA). Listeners with CIs used their sound processors and a direct audio input cable connected to the output of the soundcard and amplifier to listen to stimuli presented at a comfortable level. If their sound processor did not accommodate direct audio input (as is the case with many of the newer processors), the acoustic signal was presented to listeners with CIs through headphones (Sennheiser HD650s) placed over the processor’s microphone. For listeners with normal hearing, five sentences at each of the four degrees of time compression (no compression or 0, 20, 40, and 60%) and the four degrees of vocoding (none or unprocessed, 16 channels, 8 channels, 4 channels) were presented in a random order for a total of 80 sentences in a single block. These listeners heard four blocks with no repeated sentences for a grand total of 320 sentences (20 in each degree of time compression/number of vocoding channels condition). For listeners with CIs, 10 sentences at each degree of time compression in blocks of 40 sentences were presented in random order and without replacement. Listeners with CIs heard four blocks of 40 sentences for a grand total of 160 sentences (40 at each degree of time compression). This is twice the number of sentences heard at each degree of time compression compared to the listeners with NH, but fewer sentences overall because the listeners with NH also completed three vocoded conditions.

Listeners were asked to repeat each sentence aloud and an experimenter in the room marked which of five keywords in each sentence were correct. Listeners were encouraged to guess if they were unsure of a word. No feedback was provided during the experiment. Scoring followed the protocol outlined by Stilp et al. (2010): no penalty was imposed for guessing incorrect words, incorrect word order, or incorrect word endings as long as the pronunciation of the root was unchanged (e.g., “help” was scored as a correct response for “helped” but “drink” was scored as incorrect for “drank”). Guesses that included incorrect word endings without changing the pronunciation of the root were extremely rare.

### Analysis

Data were analyzed using generalized linear mixed effects regression modeling with a binomial distribution using the lme4 package version 1.1.27.1 (Bates et al., 2015) in R version 4.1.1 (R Core Team, 2021). These models use trial-by-trial data to predict the (log-odds) probability of a correct response. The dependent variable was the percentage of correct keywords per sentence (out of five). Amount of time compression (four levels: 0, 20, 40, and 60%), number of vocoded channels (four levels: unprocessed, 16, 8, and 4), age group (three levels: younger, middle-aged, and older), and the mean-centered standardized scores from the Symbol Search task were used as the independent variables. One model was fit to the data from the listeners with NH while a separate model was fit to the data from the listeners with CIs. This second model did not include the vocoding variable because the listeners with CIs did not listen to any vocoded sentences. A third model was fit to compare listeners with CIs and listeners with NH presented 8-channel vocoded speech–the number of channels associated with the average spectral resolution available to listeners with CIs (Friesen et al., 2001).

Mixed effects models are able to model multiple sources of random variability. This allows the models to explain more variance than simpler fixed effects models (e.g., regression, ANOVA, generalized linear models). The procedure for model building described in Hox et al. (2017) was followed. First, a model with only the random intercepts of listener and sentence was run as a baseline. Then, the independent variables related to the hypotheses were added to the model as fixed terms and interaction terms, including time compression, number of vocoded channels (for the model on the data from listeners with NH), and age group. The Symbol Search scores were not predicted to interact with any of these variables and so this variable was added only as a fixed effect (no interactions). After fitting the model with the predicted fixed effects, random slopes were added to the model. First, a maximal random effects structure was attempted. This included all possible slopes and interactions: time compression and number of vocoding channels on the listener intercept and age group, time compression, and number of vocoding channels on the sentence intercept. If it converged, each model version was compared to previous iterations using a Likelihood Ratio Test. A systematic trial of all possible combinations identified the model with the lowest Akaike Information Criterion (AIC) and the maximal random effects structure that still converged, as suggested by Barr et al. (2013). This maximal converging model then underwent stepwise backward elimination, first eliminating non-significant interaction terms until the terms that remained were either significant themselves or contributed to a significant interaction. The reduced model was presented if a Likelihood Ratio Test showed it to be a significantly better fit to the data than the maximal model.

## Results

### Listeners with normal hearing presented vocoded speech

**FIGURE 2:**
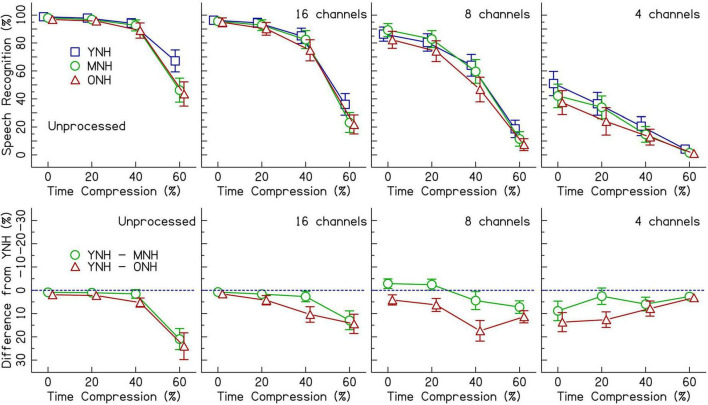
The top row shows speech recognition performance in percent correct for listeners with normal hearing (NH) in three age groups (younger, middle-aged, and older) listening to various levels of vocoding (unprocessed, 16-channel, 8-channel, and 4-channel) and four rates of time compression (0, 20, 40, and 60%). Error bars show ± 1 standard error. The bottom row shows the difference from the performance of the younger age group in percent. The dotted blue line represents the performance of the younger listeners. Data points below the line represent worse performance than the younger group. Error bars show ± 1 standard error and are based on 10,000 bootstrapped differences.

Figure 2 (top row) shows speech recognition performance for listeners with NH across increasing rates of time compression. Each panel displays the performance when listening to different CI simulations. Age-related differences are best observed in the bottom row of Figure 2. The performance of the middle-aged and older groups is plotted as a difference (in percent correct) from the performance of the younger group–represented by the dotted line. Performance was at ceiling for unprocessed speech in the 0 and 20% time-compressed conditions and declined beginning at 40% time compression. With 60% time compression, the performance of the MNH and ONH listeners declined even further. The decline in performance for the middle-aged and older listeners appears to be larger than the decline in performance for YNH listeners as shown in the top row of the figure. Similar age-related declines for recognition of time-compressed speech by MNH and ONH listeners, relative to YNH listeners, were observed when speech was vocoded with 16 and 8 channels. In the 4-channel vocoded condition, performance was at floor for 60% time-compressed speech. Overall, speech recognition performance decreased as the number of vocoder channels decreased.

Results from the generalized linear mixed effects model on the trial-by-trial data from the listeners with NH are shown in Table 2. The intercept estimate represents the predicted log-odds speech recognition performance of the YNH listener group in the 0% time-compressed and unprocessed speech condition. The other values listed in the table are the changes in performance for the given variables from the reference group (YNH) and the reference conditions (unprocessed and 0% time compression). The analysis revealed significant interactions between age group and time compression. The significance of these interactions was driven by the differences in performance at 40 and 60% time compression between the YNH group and both the MNH and ONH groups. Both of the older listener groups performed more poorly than the younger group at these degrees of time compression. To determine if there was a significant difference between the MNH and ONH groups, the model was releveled with the MNH group as the reference. There were no significant differences in performance between the MNH and ONH groups for 40 and 60% time-compressed speech or overall (all *p*’s > 0.05). In the model presented in Table 2, there were also significant main effects of vocoding compared to unprocessed speech (all *p*’s < 0.001) and one interaction between 8-channel vocoded speech and the middle-aged listener group. This interaction showed that, on average, the middle-aged group performed better than the younger group with 8-channel vocoded speech (*z* = 2.40, *p* = 0.02). There was no significant main effect of Symbol Search scores, no significant interactions between vocoding and time compression, and no significant higher-order interactions (all *p*’s > 0.05). The random intercepts of sentence and listener accounted for some of the variance in the data. Including random slopes for age group, amount of time compression, and number of vocoder channels in a maximal random effects structure as per Barr et al. (2013) improved model fit.

**TABLE 2:** Logistic mixed-effects model describing the effects of experimental variables and other predictors on speech recognition performance of listeners with NH.

Fixed effects	Log-odds estimate	SE	*z*	*p*	
(Intercept)	5.41	0.21	26.22	<0.001	***
AgeMNH	–0.23	0.27	–0.86	0.391	
AgeONH	–0.48	0.27	–1.76	0.079	
TC20	–0.53	0.08	–6.58	<0.001	***
TC40	–1.58	0.08	–19.43	<0.001	***
TC60	–4.40	0.10	–45.59	<0.001	***
Channels16	–1.54	0.12	–12.35	<0.001	***
Channels8	–3.04	0.14	–21.06	<0.001	***
Channels4	–5.49	0.18	–30.90	<0.001	***
AgeMNH × TC20	0.01	0.10	0.13	0.894	
AgeONH × TC20	–0.13	0.10	–1.27	0.205	
AgeMNH × TC40	–0.35	0.10	–3.47	<0.001	***
AgeONH × TC40	–0.49	0.10	–4.80	<0.001	***
AgeMNH × TC60	–0.96	0.12	–8.27	<0.001	***
AgeONH × TC60	–0.95	0.12	–7.91	<0.001	***
AgeMNH × Channels16	0.22	0.16	1.41	0.159	
AgeONH × Channels16	0.17	0.16	1.04	0.299	
AgeMNH × Channels8	0.45	0.19	2.40	0.017	*
AgeONH × Channels8	0.12	0.19	0.65	0.516	
AgeMNH × Channels4	–0.09	0.23	–0.41	0.680	
AgeONH × Channels4	–0.17	0.23	–0.73	0.464	

**Random effects**	**Variance**	**SD**	**Correlations**							

By-Sentence intercepts	2.58	1.61								
By-Sentence AgeMNH slopes	0.44	0.67	–0.33							
By-Sentence AgeONH slopes	0.39	0.63	–0.28	0.48						
By-Sentence TC20 slopes	0.51	0.71	–0.13	0.11	–0.06					
By-Sentence TC40 slopes	0.79	0.89	–0.40	0.11	0.06	0.49				
By-Sentence TC60 slopes	1.53	1.24	–0.63	0.17	0.21	0.24	0.68			
By-Sentence Channels16 slopes	0.90	0.95	–0.51	–0.03	–0.04	–0.17	0.18	0.17		
By-Sentence Channels8 slopes	1.37	1.17	–0.67	0.18	0.08	–0.12	0.15	0.40	0.75	
By-Sentence Channels4 slopes	2.55	1.60	–0.68	0.14	0.18	–0.10	0.18	0.42	0.61	0.74
By-Listener intercepts	0.43	0.66								
By-Listener Channels16 slopes	0.09	0.31	–0.50							
By-Listener Channels8 slopes	0.17	0.41	–0.74	0.81						
By-Listener Channels4 slopes	0.28	0.53	–0.60	0.82	0.89				

Significant fixed effects are marked with asterisks, with p-values generated by Wald z-scores. The intercept estimate represents the predicted log-odds speech recognition performance of the YNH listener group in the 0% time-compressed and unprocessed speech condition, which is used as the reference for all other conditions. The values in the first column of the Correlations reflect the correlation of that row’s variable with the intercept. The values in the second column reflect the correlation of that row’s variable with the first slope variable under the common intercept. Significance codes: “***” < 0.001; “**” < 0.01; “*” < 0.05.

### Listeners with cochlear implants

**FIGURE 3:**
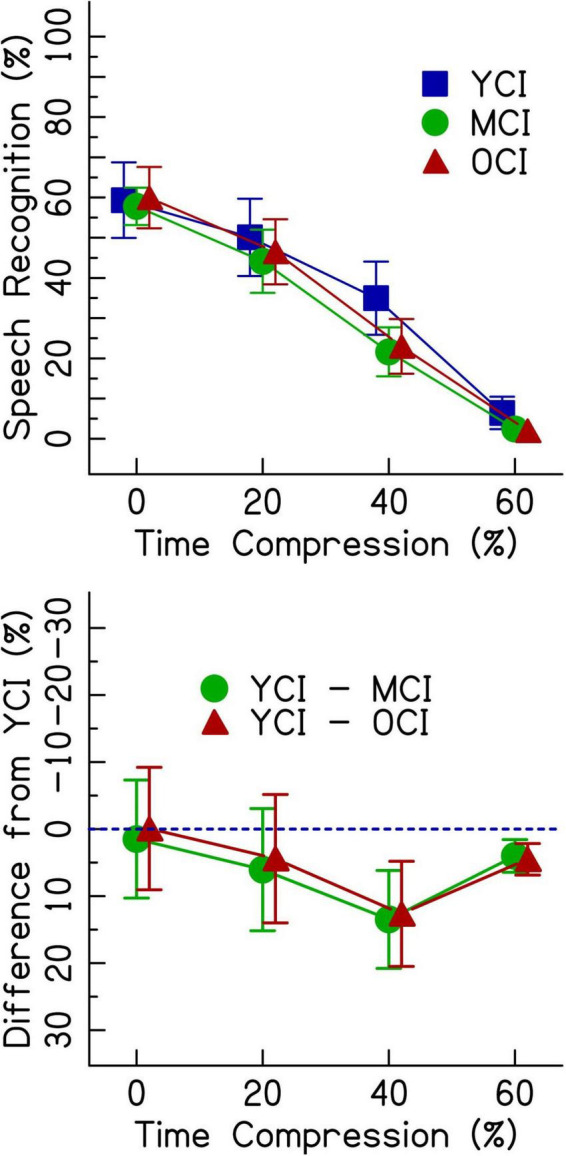
The top panel shows speech recognition performance in percent correct for listeners with CIs in three age groups (younger, middle-aged, and older) listening to four rates of time compression (0, 20, 40, and 60%). Error bars show ± 1 standard error. The bottom panel shows the difference from the performance of the younger age group in percent. The dotted blue line represents the performance of the younger listeners. Data points below the line represent worse performance than the younger group. Error bars show ± 1 standard error and are based on 10,000 bootstrapped differences and the smallest group size of 16.

Figure 3 (top panel) shows speech recognition performance for listeners with CIs. Unlike the listeners with NH, performance was not at ceiling for 0% time-compressed speech. All age groups performed with about 60% accuracy for 0% time-compressed speech. Generally, there were greater performance decrements on time-compressed speech for the MCI and OCI listeners compared to the YCI listeners. Age-related differences in performance are best observed in the bottom panel of Figure 3, where the performance of the middle-aged and older groups are shown as the difference from the performance from the younger group–represented by the dotted line. In the 60% time-compressed condition, performance was near the floor. Overall, speech recognition performance decreased as time compression increased. This follows the same general trend as was seen in the performance of listeners with NH.

Results from the generalized linear mixed effects model on the data from the listeners with CIs are shown in Table 3. The analysis revealed significant interactions between age group and time compression. The significance of these interactions was driven by the differences in performance between the YCI group and both the MCI and OCI groups in the time-compressed conditions. Recognition of 20% time-compressed speech was poorer for the MCI group compared to the YCI group (Table 3: AgeMCI × TC20, *z* = −2.14, *p* = 0.032). The performance of the OCI group was not significantly different than the YCI group at this time compression ratio (Table 3: AgeOCI × TC20, *z* = −1.79, *p* > 0.05). Recognition of 40 and 60% time-compressed speech was poorer for both the MCI and OCI groups compared to the YCI group (Table 3: AgeMCI × TC40, *z* = −3.45, *p* < 0.001 and AgeOCI × TC40, *z* = −4.27, *p* < 0.001; AgeMCI × TC60, *z* = −2.74, *p* = 0.006 and AgeOCI × TC60, *z* = −2.95, *p* = 0.003). To determine if there was a significant difference between the two older age groups, the model was releveled with the MCI group as the reference. There were no significant differences in performance between the MCI and OCI groups at either 40% time compression (*z* = −1.96, *p* > 0.05) or 60% time compression (*z* = −0.94, *p* > 0.05, analysis summary table not shown). There was no significant main effect of Symbol Search scores (*p* > 0.05).

**TABLE 3:** Logistic mixed-effects model describing the effects of experimental variables and other predictors on speech recognition performance of listeners with CIs.

Fixed effects	Log-odds estimate	SE	*z*	*p*	
(Intercept)	0.81	0.38	2.12	0.034	*
AgeMCI	–0.48	0.52	–0.93	0.354	
AgeOCI	–0.16	0.50	–0.31	0.757	
TC20	–0.60	0.11	–5.71	<0.001	***
TC40	–1.87	0.13	–13.95	<0.001	***
TC60	–4.91	0.29	–17.02	<0.001	***
AgeMCI × TC20	–0.29	0.14	–2.14	0.032	*
AgeOCI × TC20	–0.24	0.13	–1.79	0.074	
AgeMCI × TC40	–0.61	0.18	–3.45	<0.001	***
AgeOCI × TC40	–0.74	0.17	–4.27	<0.001	***
AgeMCI × TC60	–1.07	0.39	–2.74	0.006	**
AgeOCI × TC60	–1.08	0.37	–2.95	0.003	**

**Random effects**	**Variance**	**SD**	**Correlations**				

By-Sentence intercepts	1.10	1.05					
By-Sentence AgeMCI slopes	0.61	0.78	–0.43				
By-Sentence AgeOCI slopes	0.57	0.75	–0.34	0.67			
By-Sentence TC20 slopes	0.40	0.64	–0.02	0.00	–0.16		
By-Sentence TC40 slopes	0.71	0.84	–0.10	0.08	–0.06	0.30	
By-Sentence TC60 slopes	1.53	1.24	–0.37	0.20	0.05	0.28	0.49
By-Listener intercepts	2.10	1.45					
By-Listener TC20 slopes	0.05	0.22	0.09				
By-Listener TC40 slopes	0.13	0.35	–0.03	0.59			
By-Listener TC60 slopes	0.66	0.81	–0.34	0.31	0.73		

Significant fixed effects are marked with asterisks, with p-values generated by Wald z-scores. The intercept estimate represents the predicted log-odds speech recognition performance of the YCI listener group in the 0% time-compressed speech condition, which is used as the reference for all other conditions. The values in the first column of the Correlations reflect the correlation of that row’s variable with the intercept. The values in the second column reflect the correlation of that row’s variable with the first slope variable under the common intercept. Significance codes: “***” < 0.001; “**” < 0.01; “*” < 0.05.

### Comparison between listener groups

**FIGURE 4:**
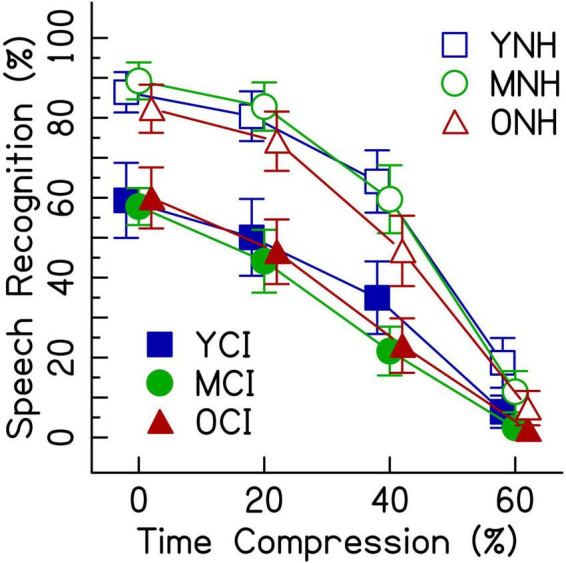
Speech recognition performance in percent correct for listeners with CIs and listeners with NH presented 8-channel noise vocoded speech. Each listening group had three age groups (younger, middle-aged, and older) and listened to four rates of time compression (0, 20, 40, and 60%). Error bars show ± 1 standard error.

Given that the average listener with a CI uses roughly eight channels of spectral resolution (Friesen et al., 2001), the 8-channel vocoding condition was chosen to compare the performance of listeners with NH to that of listeners with CIs. Figure 4 shows speech recognition scores from both listener groups. Results from the generalized linear mixed effects model comparing the performance of the two groups (Table 4) show a main effect of listener group (Table 4: ListenerGroupCI, *z* = −7.05, *p* < 0.001), with listeners with CIs generally performing more poorly than listeners with NH presented with vocoded speech. There were also significant interactions between age groups (middle-aged and older) and the greater degrees of time compression (40 and 60%) (all *p*’s < 0.001). These interactions indicate that for both listener groups, the middle-aged and older listeners’ recognition of both the 40 and 60% time-compressed speech was poorer than that of the younger listeners at those time-compression ratios. In addition, there were significant interactions between listener group and all degrees of time compression (all *p*’s < 0.01). These interactions indicate that for all age groups, listeners with CIs recognize time-compressed speech more poorly than listeners with NH. However, these interactions should be interpreted with caution because of the floor effects present in the data and because performance was not matched between the two listener groups in the 0% time-compressed condition. There were no significant listener group × age group interactions (all *p*’s > 0.05). This lack of an interaction between listener group and age group suggests that the age-related differences in recognition of time-compressed speech (i.e., between younger, middle-aged, and older listeners) may be similar in both CI and NH listener groups. There were also no three-way interactions between degree of time compression, age group, and listener group, reinforcing the notion that the interaction between age group and degree of time compression may affect listeners with CIs and with NH in a comparable way and the interaction between listener group and degree of time compression may also be equivalent across all age groups.

**TABLE 4:** Logistic mixed-effects model describing the effects of experimental variables and other predictors on recognition performance for time-compressed speech by listeners with CIs and listeners with NH presented a simulation of CI-processed speech (8-channel noise vocoding).

Fixed effects	Log-odds estimate	SE	*z*	*p*	
(Intercept)	2.36	0.25	9.55	<0.001	***
AgeMiddleAged	–0.06	0.29	–0.21	0.837	
AgeOlder	–0.20	0.29	–0.68	0.497	
ListenerGroupCI	–1.68	0.24	–7.05	<0.001	***
TC20	–0.37	0.10	–3.57	<0.001	***
TC40	–1.46	0.13	–11.60	<0.001	***
TC60	–4.26	0.20	–21.47	<0.001	***
AgeMiddleAged × TC20	–0.21	0.10	–2.01	0.044	*
AgeOlder × TC20	–0.17	0.10	–1.65	0.098	
AgeMiddleAged × TC40	–0.60	0.14	–4.35	<0.001	***
AgeOlder × TC40	–0.70	0.14	–5.14	<0.001	***
AgeMiddleAged × TC60	–1.02	0.24	–4.29	<0.001	***
AgeOlder × TC60	–1.11	0.24	–4.67	<0.001	***
ListenerGroupCI × TC20	–0.29	0.09	–3.09	0.002	**
ListenerGroupCI × TC40	–0.38	0.12	–3.25	0.001	**
ListenerGroupCI × TC60	–0.60	0.21	–2.89	0.004	**

**Random effects**	**Variance**	**SD**	**Correlations**					

By-Sentence intercepts	1.19	1.09						
By-Sentence AgeMiddleAged slopes	0.41	0.64	–0.32					
By-Sentence AgeOlder slopes	0.49	0.70	–0.32	0.63				
By-Sentence ListenerGroupCI slopes	0.72	0.85	–0.51	0.06	0.09			
By-Sentence TC20 slopes	0.34	0.58	0.06	–0.06	–0.14	–0.09		
By-Sentence TC40 slopes	0.64	0.80	–0.16	0.00	–0.06	0.05	0.47	
By-Sentence TC60 slopes	1.25	1.12	–0.34	0.04	0.08	0.14	0.14	0.58
By-Listener intercepts	1.24	1.12						
By-Listener TC20 slopes	0.03	0.18	0.08					
By-Listener TC40 slopes	0.14	0.38	–0.14	0.74				
By-Listener TC60 slopes	0.51	0.71	–0.29	0.27	0.71			

Significant fixed effects are marked with asterisks, with p-values generated by Wald z-scores. The intercept estimate represents the predicted log-odds speech recognition performance of the YNH listener group in the 0% time-compressed speech condition, which is used as a reference for all other conditions. The values in the first column of the Correlations reflect the correlation of that row’s variable with the intercept. The values in the second column reflect the correlation of that row’s variable with the first slope variable under the common intercept. Significance codes: “***” < 0.001; “**” < 0.01; “*” < 0.05.

## Discussion

The results of the current study provide insight into the interactions between multiple types of distortion for older listeners with CIs. This study replicated the known separate effects of the distortion of a CI processor, rapid or time-compressed speech, and aging in the central auditory processing system. The results further showed significant interactions between higher degrees of time compression and age. This finding supports the first hypothesis, which was that there would be an age group × degree of time compression interaction for both CI and NH listener groups, specifically that larger age-related decreases would be observed with greater degrees of time compression. The results also showed that scores from a measure of general processing speed did not improve model fit significantly for either listener group (Tables 2, 3), indicating that this measure did not contribute to listener performance. This finding did not support the second hypothesis, which was that faster cognitive processing speed would be predictive of better performance in recognizing time-compressed speech.

### Effects of cochlear implant processing and age

Previous research has shown auditory temporal processing deficits in older listeners compared to younger listeners (e.g., Gordon-Salant and Fitzgibbons, 1993; Gordon-Salant et al., 2006, 2007), as well as deficits in understanding time-compressed speech by listeners with CIs (e.g., Fu et al., 2001; Ji et al., 2014). The current study was designed to challenge the auditory temporal processing abilities of the listeners and reveal how age-related temporal processing deficits might impact the speech recognition of OCI listeners and/or ONH listeners presented a simulation of CI-processed speech. When speech was presented at a typical rate (the 0% time-compressed conditions) to listeners with NH, speech understanding performance decreased as the number of vocoder channels decreased. However, there were no significant age group effects and no significant age group × number of vocoded channels interactions. While Figure 2 may appear to show a difference between age groups in the 0% time-compressed 4-channel condition, once the random effects of sentences and listeners were added to the model, the difference between age groups in that condition was no longer significant. This indicates that listeners with NH are affected similarly across age groups by the degree of spectral distortion. Listeners with CIs also show no significant differences in performance between age groups when speech is presented at 0% time compression.

The speech understanding scores differ between listeners with NH presented vocoded speech and listeners with CIs (Figure 4). Performance of listeners with CIs was lower than that of the listeners with NH presented with 8-channel vocoded speech and higher than that of the listeners with NH presented with 4-channel vocoded speech. Perhaps a simulation of 6-channel vocoded speech would have better matched performance between the two listener groups. Alternatively, one-to-one matching of listeners by age and performance could be done (Bhargava et al., 2016; Tinnemore et al., 2020). Choosing a simulation that perfectly matches performance, however, is complicated by the differences in experience listening to spectrally degraded speech, since performance can change over exposure time (e.g., Rosen et al., 1999; Davis et al., 2005; Smalt et al., 2013; Waked et al., 2017). Had the listeners with NH received more practice with vocoded speech than the short training session we provided, the size of the group effects and interactions could have changed.

In theory, the spiral ganglia in the cochlea are the main part of the peripheral auditory system that remain vulnerable to age-related changes and affect speech understanding in listeners with CIs. There are age-related differences in spiral ganglia survival, even in ears with no hair cell loss (Makary et al., 2011). Measures of neural survival in the cochlea, such as electrically evoked compound action potentials, show promise in explaining some of the variance in listener performance with a CI across age groups (e.g., Jahn and Arenberg, 2020; Shader et al., 2020a; Jahn et al., 2021). These measures can provide objective evidence toward the strength or weakness of the electrode-to-neuron interface, which affects the integrity of the signal received by the brain but cannot measure any potential central or cognitive changes. Another factor that affects the electrode-to-neuron interface and speech recognition in listeners with CIs is the placement of the electrode arrays as determined by CT scans (e.g., Berg et al., 2020, 2021). Better simulations that could help match performance between the two listener groups are likely dependent on the stimuli or other individual factors such as array type, insertion depth, and array placement (e.g., Croghan et al., 2017; Berg et al., 2019, 2020). A simulation that accounts for these factors, such as the SPIRAL vocoder (Grange et al., 2017), would allow for more valid comparisons between listener groups.

### Effects of time compression and age

As expected, performance decreased with increasing time compression for both listeners with CIs and listeners with NH (Figures 2, 3). The interaction between greater degrees of time compression and age group in each of the three analyses indicates that the middle-aged and older groups recognize time-compressed speech with less accuracy than the younger listeners, regardless of whether they are listening through a CI or to a CI simulation. These results are consistent with previous studies that showed significant interactions between age group and amount of time compression for unprocessed (i.e., non-vocoded) sentences in listeners with NH (e.g., Gordon-Salant and Fitzgibbons, 1993; Tun, 1998) and listeners with age-related hearing loss (Gordon-Salant and Fitzgibbons, 1993).

The current results expand upon previous studies that were conducted with listeners who use CIs. Ji et al. (2013) presented results from 10 listeners with CIs who ranged in age from 24 to 81 years old (*M* = 65.2) and who were presented IEEE sentences that had been 50% time compressed. There was a significant effect of time compression on the speech recognition of these listeners with CIs. The current study expanded the number of listeners and included 58 listeners with CIs who were assigned to one of three age groups with >15 listeners/group (Table 1). This allowed the factor of age group to be analyzed as a possible source of variance in listeners with CIs. The current study also varied the degree of time compression and showed interactions between time compression ratio and age group in listeners with CIs, such that the MCI and OCI listeners’ recognition of 40 and 60% time-compressed speech was poorer than that of YCI listeners. In conditions with time-compressed speech, the performance of the middle-aged listeners with CIs and with NH presented vocoded speech was consistent with that of the older listeners, rather than appearing at an intermediate range between the younger and older listeners. This suggests that the effects attributed to age are likely affecting the performance of listeners as young as 50 years old (or younger). Together, these results demonstrate that listening to rapid or time-compressed speech through a CI or through spectral degradation similar to that imposed by a CI severely challenges the speech recognition of middle-aged and older listeners.

### Effects of cognitive processing speed and age

Contrary to the second hypothesis, cognitive processing speed did not predict recognition of time-compressed speech (Tables 2, 3), and therefore its role in understanding time-compressed speech remains an open question. It was assumed that a measure of cognitive processing speed would affect recognition of time-compressed speech based on previous research (e.g., Wingfield et al., 1985; Dias et al., 2019). Wingfield et al. (1985) showed that word recognition accuracy decreased more for older listeners as speech rate increased than it did for younger listeners and argued that this was evidence of a difference in processing speed. Dias et al. (2019) used the Connections Test (Salthouse et al., 2000) and showed that a derived measure of perceptual processing speed mediated age-related variability in recognition of time-compressed speech. In the current study, non-auditory processing speed was measured directly using the Symbol Search subtest of the WAIS (Wechsler, 1955). Given the current non-significant result, it is possible that general cognitive processing speed may not play a strong role in the recognition of time-compressed speech. Alternatively, it is possible that the measure of processing speed chosen for this study was not sensitive enough to capture subtle cognitive deficits in auditory processing speed that might influence the ability to recognize rapid speech. Future studies should consider alternative measures of cognitive processing speed.

Other cognitive abilities have been shown to affect performance on sentence recognition tasks. Specifically, working memory correlates with measures of auditory temporal processing, including time-compressed speech (e.g., Vaughan et al., 2006; Humes et al., 2022). Working memory also correlates with performance on distorted speech (e.g., speech in noise) for listeners with hearing impairment (e.g., Rönnberg et al., 2010; Rudner et al., 2011; Zekveld et al., 2013; Füllgrabe et al., 2015). Therefore, another approach would be to assess other cognitive abilities, such as working memory, as predictors of performance on time-compressed speech recognition tasks.

Yet another approach would be an assessment of neural processing of time-compressed speech. Older adults show reduced neural synchrony to normal-rate speech in speech-evoked responses from the brainstem (e.g., Anderson et al., 2012) to the cortex (e.g., Tremblay et al., 2002; Goossens et al., 2016) compared to younger adults. This reduced neural synchrony has been hypothesized to contribute to older adults’ difficulties understanding speech in noise (e.g., Aubanel et al., 2016). In the cortex, the timescale of neural oscillations may be related to linguistic processing (e.g., Greenberg, 1999; Ghitza and Greenberg, 2009; Giraud and Poeppel, 2012; Peelle and Davis, 2012) since individual neurons can adapt to small changes in the rate of speech (e.g., Lerner et al., 2014). In addition, neural oscillations may adapt better to naturally rapid speech than to time-compressed speech (e.g., Hincapié Casas et al., 2021). A measure of the accuracy of a listener’s neurons to track acoustic modulation in speech may better predict performance on time-compressed speech than measures of cognition.

### Other limitations and future directions

Listeners in the current study with NH were designated as having NH based on thresholds at octave frequencies up to 4,000 Hz. The speech stimuli used in the study contained frequencies above 4,000 Hz. Both the MNH and the ONH groups had significantly poorer thresholds than the younger listeners at 8,000 Hz [Figure 1; MNH vs. YNH: *t*(27) = −4.7, *p* < 0.001; ONH vs. YNH: *t*(27) = −5.4, *p* < 0.001]. These differences in hearing thresholds at a frequency outside the range used as criteria for the study could have driven some of the performance differences attributed to age.

Listeners in the CI group included those who were born with acoustic hearing and later acquired significant hearing loss, as well as those who were born with little or no acoustic hearing. Most of this latter group were in the YCI group. As a group, they had lower performance overall, likely due to their altered experience learning language through the distortions of a CI processor (e.g., Kirk and Hill-Brown, 1985; Tye-Murray et al., 1995). The etiologies of hearing loss in the younger age group are also distinct from those in the middle-aged and older groups. While etiology has been shown to affect speech recognition outcomes overall in listeners with CIs (Blamey et al., 2013), it is unknown whether the etiology of hearing loss affects the ability to recognize time-compressed speech.

Future studies might benefit from purposefully recruiting listeners with a more uniform distribution of ages. Better matching of ages and performances across listener groups would increase statistical power and might allow interactions between listener groups and experimental factors to reach significance. In the current study, the effects of time compression and age were not significantly different in listeners with NH or CIs. Alternatively, there could be other latent variables in the demographics or listener characteristics that could contribute to variation in temporal processing abilities between listening to vocoded speech and listening with a CI (e.g., years of education, history of noise exposure, years of musical training).

The current study did not directly measure basic non-speech central auditory temporal processing abilities of the listeners, leaving their potential contributions to be inferred. The current study also did not eliminate the possibility that age-related central changes could be caused by peripheral hearing loss that might have occurred before implantation in several listeners in the OCI group. The cause of central auditory deficits cannot be determined solely from performance on a perceptual task, such as that described in the current study. The explanatory power of central auditory processing or electrophysiology measures compared to cognitive predictors could provide additional insight into the source, or combination of sources, of the age-related deficits observed in understanding time-compressed speech.

### Summary

This study demonstrated that the deficits in speech recognition of older listeners for time-compressed speech may be primarily affected by age-related declines in central auditory processing rather than solely related to peripheral age-related hearing loss. The findings did not support the notion that the cognitive domain of processing speed contributes to age-related declines in recognition of time-compressed speech. While performance was affected by the degradation introduced to the speech signal by the CI sound processor and the CI simulations, there was no difference between age groups for normal-rate speech. The interactions between age group and time compression highlight the challenge of understanding rapid speech, especially for older listeners. The older and middle age groups showed similar performances, regardless of the mode of listening (acoustic or with a CI), indicating that potential age-related differences in central auditory processing may affect performance by adults prior to 65 years of age.

The similarities in the effects of time compression on both listeners with CIs and listeners with NH suggest a common source of the deficits associated with older listeners’ recognition of time-compressed speech. Even without vocoding, there was a significant effect of greater degrees of time compression on the speech recognition performance of ONH listeners. Given the vast differences in the acoustic signal between unprocessed speech and noise-band vocoded speech, and the similarities in performance between listeners with NH and those with CIs, it appears that the oft-reported deficits in recognition of time-compressed speech exhibited by ONH acoustic-hearing listeners may be at least partially explained by central auditory processing abilities.

## Data availability statement

The original contributions presented in this study are publicly available. This data can be found here: https://osf.io/zvb7u/, 10.17605/OSF.IO/ZVB7U.

## Ethics statement

All procedures were conducted with the informed consent of the listeners and were approved by the Institutional Review Board of the University of Maryland. Listeners were compensated for their time and participation.

## Author contributions

AT collected the data, performed the analyses, and drafted the manuscript. LM helped design the study, collected the data, and wrote an early draft. SG-S helped design the study and contributed significantly to manuscript preparation. MG designed the study, programmed the experiment, oversaw data collection, obtained funding for the study, and provided extensive edits to all versions of the manuscript. All authors contributed to the article and approved the submitted version.
